# Methemoglobinemia presenting in a circumcised baby following application of prilocaine: a case report

**DOI:** 10.1186/1752-1947-4-49

**Published:** 2010-02-10

**Authors:** Hatice Ozdogan, Selcan Osma, Gozde B Aydin, Avni Dinc, Gulten Ozgun

**Affiliations:** 1 Department of Anesthesiology and Reanimation, Dışkapı Yıldırım Beyazıt Training and Research Hospital, Ulus Dışkapı, Ankara, Turkey

## Abstract

**Introduction:**

Local anesthesia with prilocaine has become a routine part of ambulatory circumcision procedures. Methemoglobinemia is a rare but potentially lethal complication of local anesthetics.

**Case presentation:**

We report the case of a 40-day-old Turkish boy who presented with cyanosis after receiving local anesthesia with prilocaine. His methemoglobin level revealed severe methemoglobinemia (methemoglobin = 44%). His cyanosis resolved after intravenous administration of methylene blue.

**Conclusion:**

Although the association between prilocaine use and methemoglobinemia has generally restricted the use of prilocaine in babies, it is still widely used in ambulatory procedures, especially during circumcision in the neonatal period. Prilocaine should not be used in babies who are less than 3 months old because of the risk of methemoglobinemia; other local anesthetics may be used for this age group. Furthermore, general anesthesia by mask ventilation may be favored for babies less than 3 months of age instead of local anesthetics.

## Introduction

Circumcision is the surgical removal of the foreskin of the glans penis. Newborns undergoing circumcision demonstrate objective, measurable evidence of pain, yet the procedure is often performed without analgesia. Newborn circumcision is reported to be the most common elective surgical procedure performed on infants in the United States [1]. There is still significant controversy regarding the benefits and risks of newborn circumcision. In recent years, a number of authors have reported medical benefits of newborn circumcision including a decrease in the number of infants with urinary tract infections, protection against penile cancer, protection against HIV infection and protection against transmission of human papilloma virus [1].

Anesthesia is not routinely administered for neonatal circumcision for a variety of reasons, for example, the relatively short duration of the intervention, the perceived lack of importance of the pain, and concerns regarding possible toxicity of the medication [2]. Administration of anesthesia attenuates acute circumcision pain and can prevent or reduce some of the short-term and long-term behavioral effects [3]. For many years, it has been suggested that newborn infants or neonates were unable to feel pain because of their immature nervous system. Nowadays, it is commonly accepted that newborn infants can feel pain and should be treated with that taken into consideration [4].

Local anesthesia infiltration by injection is considered to be the most effective method of analgesia for circumcision. The method most commonly used to administer the anesthetic is the dorsal penile nerve block (DPNB), first described in 1978 [3]. Local anesthesia with prilocaine has become a routine part of ambulatory circumcision procedures [5]. Methemoglobinemia is a rare but potentially lethal complication of local anesthetics. The iron in hemoglobin is normally in the reduced, ferrous state (Fe^2+^), which is essential for its oxygen-transporting function. Under physiological conditions, there is a slow, constant loss of electrons to release oxygen and the ferric (Fe^3+^) form combines with water, producing methemoglobin. The predominant intracellular mechanism for the reduction of methemoglobin is cytochrome b5, and only 1 to 2% of hemoglobin is normally in the ferric state. As methemoglobin levels increase, oxygen delivery to tissues is impaired and cellular hypoxia develops [6]. Infants are particularly vulnerable to hemoglobin oxidation because their cytochrome b5 reductase level is approximately 50% of the adult value [6]. We report a patient with prilocaine-induced acquired methemoglobinemia and discuss the use of intravenous methylene blue treatment.

## Case presentation

A 40-day-old Turkish boy weighing 4000 g was admitted to the emergency department with cyanosis. On admission, no abnormality was detected other than central and peripheral cyanosis and anxiety. His medical history was unremarkable; he was born full term and had no perinatal problems. The history obtained from his parents revealed that he had undergone a circumcision procedure with local anesthesia, and cyanosis had begun 1.5 hours after the procedure. A dorsal nerve block with 1.5 mg/kg prilocaine (Citanest 2%; Astra-Zeneca) was applied during circumcision. His hemoglobin level was 11.9 g/dl and his white blood cell count was 11,200/mm^3^. The arterial blood gas analysis revealed pH 7.28, PO_2 _85 mmHg, and oxygen saturation 78%. Electrophoresis revealed methemoglobin levels of 44%. 100% oxygen was given but the patient did not respond to supplemental oxygen. He was diagnosed as having methemoglobinemia; underlying erythrocyte-methemoglobin reductase deficiency and glucose-6-phosphate dehydrogenase deficiency were ruled out and 1 mg/kg methylene blue was given intravenously. His cyanosis resolved in 60 minutes and the methemoglobin fraction regressed to 4% in 60 minutes and oxygen saturation increased to 92%. The methemoglobin fraction regressed to 1.5% in 24 hours. His cyanosis disappeared completely after 120 minutes.

## Discussion

Circumcision is widely used in Turkey because of religious tradition. Newborn babies are particularly susceptible to methemoglobinemia as it is related to high levels of fetal hemoglobin, which is more readily oxidized to the ferric state than is adult hemoglobin. Also the transient deficiency of cytochrome b5 reductase enzyme activity that persists for the first 3 to 4 months of life favors the development of methemoglobin in neonates [7].

Methemoglobinemia results from oxidation of ferrous iron (Fe^2+^) to ferric iron (Fe^3+^), and renders the hemoglobin molecule unavailable for oxygen transport, resulting in potentially life-threatening hypoxemia. Under physiological conditions, methemoglobin reduction is accomplished mainly by red cell nicotinamide adenine dinucleotide (NADH)-cytochrome b5 reductase (NADH-methemoglobin reductase) thus ensuring that there are insignificant amounts of methemoglobin in the circulating blood. This enzyme pathway is immature in neonates, therefore this disorder may be triggered by oxidation agents such as anesthetics used in minor surgical procedures such as circumcision [8,9]. The local anesthetic prilocaine (amide group) is a popular choice for penile blockade in circumcision owing to its short onset time and low incidence of cardiac and central nervous system toxicity. However, prilocaine is the most potent methemoglobin-forming local anesthetic [7].

Cyanosis is the first clinical event when methemoglobin levels reach ≥ 10%, but symptoms of hypoxemia and diminished oxygen transport do not appear until levels increase to 30 to 40%. Levels >70% may cause death [7]. Seizures, cardiovascular collapse and coma are seen with higher methemoglobin fractions [6]. Methemoglobinemia should be considered in differential diagnosis of a child with cyanosis [5].

Pulse oximetry is inaccurate and unreliable in patients with high methemoglobin fractions. Pulse oximetry measures light absorbance at two wavelengths (660 nm and 940 nm). Pulse oximeters do not detect methemoglobin and the distinction of hypoxia and methemoglobin is not possible, which may give a false impression of patient oxygenation. A low oxygen saturation by pulse oximetry measured in patients with normal arterial blood gases can be an indication of methemoglobinemia. The exact measurement is only possible using a CO-oximeter [10].

For methemoglobinemia caused by drug exposure, the traditional first-line treatment consists of an infusion of methylene blue, the action of which depends on the availability of reduced nicotinamide adenine dinucleotide phosphate (NADPH) within red blood cells. After an acute exposure to an oxidizing agent, treatment should be considered when methemoglobin reaches 30% in an asymptomatic patient and 20% in a symptomatic patient. Patients with anemia or cardiorespiratory problems should be treated at lower levels of methemoglobin. Methemoglobinemia due to methemoglobin M does not respond to ascorbic acid or methylene blue. Methylene blue (1% solution 1-2 mg/kg) is an oxidant, its metabolic product leucomethylene blue is a reducing agent. Therefore, large doses of methylene blue may result in higher levels of methylene blue than of leucomethylene blue, which will result in hemolysis and, paradoxically, methemoglobinemia in patients with glucose-6-phosphate dehydrogenase (G6PD) deficiency [11]. In addition, patients with G6PD deficiency may not produce sufficient NADPH to reduce methylene blue to leucomethylene blue; thus, methylene blue therapy may be ineffective in these patients [11].

Methemoglobinemia in neonates has been reported after prilocaine penile ring blocks for circumcision and also occasionally after the use of EMLA (eutectic mixture of lidocaine and prilocaine) cream [12]. There are multiple reducing pathways that maintain levels of methemoglobin. Although the predominant intracellular mechanism for the reduction of methemoglobin is cytochrome b5, adult levels of the enzyme are reached by 2 to 3 months of age. Ascorbic acid (300 mg/kg) is a potent antioxidant and reducing agent. It directly reduces methemoglobin but the rate is too slow for it to be effective [13]. Although under physiological conditions, ascorbic acid-induced methemoglobin reduction is less important than reduction by the NADP-dependent methemoglobin reductase system, under methemoglobinemic conditions, treatment with ascorbic acid is possible. Ascorbic acid is widely available and due to its effect on scavenging free radicals, it can be recommended as an alternative.

Guay [14] summarized all episodes of local anesthetic-related methemoglobinemia found in the medical literature. He found 242 episodes (40.1% published in the year 2000 or after) concerning local anesthetics and methemoglobinemia. He reported that plain prilocaine may induce clinically symptomatic methemoglobinemia in children older than 6 months at doses exceeding 2.5 mg/kg. Also in adults, the dose of prilocaine should be kept lower than 5.0 mg/kg, which is reduced to 3.2 mg/kg in the presence of renal insufficiency and to 1.3 mg/kg if other oxidizing drugs are used concurrently. He concluded that prilocaine should not be used in children younger than 6 months, in pregnant women, or in patients taking other oxidizing drugs. The dose should be limited to 2.5 mg/kg.

## Conclusions

As methemoglobinemia is a rare but potentially lethal complication of local anesthetic, physicians should identify patients who are at increased risk of developing methemoglobinemia before administering local anesthetic. Prilocaine should not be used in infants less than 3 months of age because of the risk of methemoglobinemia. Methylene blue is the treatment of choice, but ascorbic acid can also be considered in treatment [5]. General anesthesia by mask ventilation rather than local anesthetic may be favored in infants younger than 3 months old.

## Abbreviations

DPNB: dorsal penile nerve block; EMLA: Eutectic Mixture of Lidocaine and Prilocaine; G6PD: glucose-6-phosphate dehydrogenase; NADH: nicotinamide adenine dinucleotide; NADPH: nicotinamide adenine dinucleotide phosphate.

## Consent

Written informed consent was obtained from the patient's parents for publication of this case report and any accompanying images. A copy of the written consent is available for review by the Editor-in-Chief of this journal.

## Competing interests

The authors declare that they have no competing interests.

## Authors' contributions

OH and OS performed patient care, analyzed and interpreted the patient data. AGB was a major contributor in writing the manuscript. DA and OG have given the final approval of the manuscript. All authors read and approved the final manuscript.
